# The NIHSS-Plus: Improving Cognitive Assessment with the NIHSS

**DOI:** 10.3233/BEN-2009-0259

**Published:** 2010-06-10

**Authors:** Rebecca F. Gottesman, Jonathan T. Kleinman, Cameron Davis, Jennifer Heidler-Gary, Melissa Newhart, Argye E. Hillis

**Affiliations:** Johns Hopkins University School of MedicineDepartment of NeurologyBaltimoreMDUSA

**Keywords:** Stroke, stroke scales, diffusion-weighted imaging, neglect, diagnosis and treatment of acute stroke (ischemia)

## Abstract

*Background:* The National Institutes of Health Stroke Scale (NIHSS) has been criticized for limited representation of cognitive dysfunction and bias towards dominant hemisphere functions. Patients may therefore receive a low NIHSS score despite a fairly large stroke. A broader scale including simple cognitive tests would improve the clinical and research utility of the NIHSS.

*Methods:* We studied 200 patients with acute non-dominant hemispheric stroke who underwent cognitive testing and had MRI with diffusion-weighted imaging (DWI) within 5 days of presentation. We measured DWI volumes and retrospectively calculated NIHSS scores. We used linear regression to determine the role of selected cognitive tests, when added to the NIHSS, in predicting DWI volume.

*Results:* The NIHSS predicted DWI volume in a univariate analysis, as did total line cancellation and a visual perception task. In a multivariate model, using log-transformed variables, the NIHSS (*p* = 0.0002), line cancellation errors (*p* = 0.02) and visual perception (*p* = 0.004) each improved prediction of total infarct volume.

*Conclusion:* The addition of line cancellation and visual perception tasks significantly adds to the model of NIHSS alone in predicting DWI volume. We propose that these two cognitive tests, which together can be completed in 2–3 minutes, could be combined with the NIHSS to create an “NIHSS-plus” that more accurately represents a patient’s ischemic tissue volume after a stroke. This scale requires further validation in a prospective study.

## 1. Background

The National Institutes of Health Stroke Scale (NIHSS) was designed primarily as a research tool [1], and is also used in acute clinical practice. However, it has been criticized for its limited representation of cognitive dysfunction and bias towards the functions of the dominant hemisphere (language). Two patients with similarly sized strokes in contralateral hemispheres may receive very different scores [2–4]. Patients with primarily subcortical strokes have stronger associations between NIHSS score and outcome [4]; those with predominantly cognitive dysfunction due to cortical stroke may receive a lower NIHSS score despite a large, disabling stroke [2,5]. A low NIHSS may lead a clinician to withhold tPA or other acute treatment, or may restrict a patient from enrollment in a clinical trial. Right-hemispheric stroke patients are less likely to receive tPA than left-hemispheric stroke patients [6].

Previous studies have suggested that tests of neglect may have stronger associations than the NIHSS with volume of neuroimaging abnormality and tissue dysfunction [7]. A broader scale that includes a few simple cognitive tests, particularly assessing neglect, could improve the measurement of outcome and selection of candidates for tPA and clinical trials. The purpose of this retrospective study was to find 1–2 cognitive tests representing right hemispheric function that, when combined with the NIHSS, would improve its representation of dysfunctional tissue and strengthen the association between the scale and infarct size in patients,

ISSN 0953-4180/09,10/$17.50 © 2009/2010 – IOS Press and the authors. All rights reserved particularly for patients with nondominant ischemic events.

## 2. Methods

### 2.1. Subjects

Subjects were a consecutive series of patients with acute nondominant stroke symptoms who were admitted to Johns Hopkins Hospital between 1999 and 2006 and met the following inclusion criteria: (1) able to provide informed consent or indicate a family member who provided informed consent; (2) no contraindication to MRI (e.g., implanted ferrous metal); (3) no prior diagnosis of neurological disease, significant hearing loss, or blindness; (4) no hemorrhage on admission CT; (4) neuroimaging with diffusion-weighted imaging (DWI) MRI and completion of at least part of the neglect battery within 24 hours of admission (within 5 days, but usually within 24 hours, of symptom onset). We also excluded patients who had left hemispheric or infratentorial acute infarcts on DWI MRI, or no infarct on DWI, to assure that any associations would be as direct as possible. All patients gave informed consent using forms approved by the Institutional Review Board.

### 2.2. Testing

Patients were administered a part of or the entire neglect battery by technicians who were blind to the imaging data. The battery included: (1) oral reading of 30 words and 5 sentences; (2) copying the "Ogden scene" [8]; (3) copying an analog clock; (4) line bisection (drawing a short vertical line at the middle of a horizontal line); (5) line cancellation (crossing out all the lines on a page) [9]; (6) visual extinction, and (7) tactile extinction. Percent errors were recorded. There were 2 scores for the visual extinction test: (a) percent errors in detecting finger wiggling in the contralateral visual field over 12 trials of simultaneous bilateral stimulus presentation (hereafter called "visual perception") and (b) the ratio of errors in detecting finger wiggling alone in the contralesional field to errors detected in finger wiggling in the contralesional field during bilateral stimulation ("visual extinction"). The first score is referred to as "visual perception" because errors can be due to either left homonymous hemianopsia or neglect. Previous studies have shown that distinguishing left homonymous hemianopsia and neglect requires visual evoked responses, since patients with severe neglect fail to respond to visual stimuli (even movement) in the contralesional visual field, even with unilateral stimulation [10]. Similarly, there were 2 measures of tactile extinction/ sensation but with light touch stimulus presented unilaterally or bilaterally. Some tests were not given to some patients because of either inability to read (for oral reading), patient unwillingness to continue, examination time cut short due to other patient commitments (e.g. diagnostic tests), or inability to understand the directions.

The NIHSS score was calculated for each patient by chart review. For some patients the NIHSS score was recorded in the chart upon admission, whereas for others we calculated the score based on the previously validated retrospective technique [11].

### 2.3. Volumetric analysis

Volumetric analysis was performed manually, without knowledge of the patient's clinical status, with the assistance of ImageJ software [12]. Volumes of infarct or dense ischemia (bright on DWI, dark on corresponding ADC maps) were traced on individual slices and calculated based on the slice thickness, and were recorded in cubic centimeters. If subjects had multiple areas of infarct, each of these lesions was measured and volumes were summed for each patient, leading to a single total acute infarct volume per participant.

### 2.4. Statistical analysis

We primarily analyzed data from patients with right hemisphere stroke because the utility of the NIHSS has been primarily criticized for these patients. Stata version 8.0 for Macintosh was used for all analyses [13]. For tests with less than 25% missing data, we performed univariate analyses including Spearman's correlations and simple linear regression to assess the relationship between each test (including NIHSS score) and DWI volume. For those cognitive tests with association by univariate analysis, we included them in a multiple linear regression model, using step-wise backwards linear regression (goal p-value $< 0.1$ per variable) along with clinical choices (selecting tests feasible to administer in an emergency room setting) to come to a final model. For this final model we log-transformed both the dependent variable (DWI volume) and some of the independent variables (cognitive test scores), given the skewed distribution of the data, to improve heteroscedasticity and improve normality of the residuals. For the cognitive test scores, we added 1 before transformation because of zero values. We used likelihood ratio testing to determine the level of significance of each variable in the model.

To help with interpretation of the results, we performed a multivariate regression with non-transformed data, but used bootstrapping to improve robustness of our estimates. We used bootstrapping with 1000 repetitions to validate the final multivariate model. This technique did not provide the primary estimates or standard errors, but provided regression coefficients for each of the tests with which to create a composite NIHSS-plus score: we converted these non-transformed regression equation coefficients to actual point values corresponding to the scale of the NIHSS score. We used 30 points as the maximum NIHSS given our patient sample (all right hemispheric lesions; the maximum score in our sample was a 25) to convert scores on the selected tasks to point values that correspond with the NIHSS score. We used the ratio of the regression coefficients to determine appropriate point values of each test, to create an NIHSS-plus score for each subject.

## 3. Results

### 3.1. Patient characteristics

Of 329 potential patients who met our inclusion criteria, 10 were excluded because 6 were missing $> 75\%$ of tests and 4 had intracerebral hemorrhage on MRI. Of the remaining patients with DWI imaging at the same time as a neglect battery, results were primarily analyzed for 200 with right hemisphere stroke (119 were excluded: 60 had TIAs's, 29 had left-hemispheric stroke, and 30 had some infratentorial DWI lesion). Characteristics of these 200 right-hemispheric stroke patients are shown in Table 1. The median volume of infarct was 8.1 cc (range 0.03–258.3 cc).

### 3.2. Tests correlated with volume of DWI abnormality

Tests that had highest Spearman's correlations with measured DWI volume were copy scene (rho = 0.48), line cancellation (rho = 0.43) and visual perception (rho = 0.44). We further explored the relationships between these tests and DWI volume in our regression analyses.

The natural log of NIHSS independently predicted natural log of DWI volume in a univariate analysis ($p < 0.0001$), as did the log-transformed line cancellation ($p < 0.0001$) and non-transformed visual perception percent errors ($p < 0.0001$). In a multivariate model combined with log-transformed NIHSS ($p = 0.0002$), log-transformed line cancellation errors ($p = 0.02$) and visual perception percent errors ($p = 0.004$) each improved prediction of log-transformed total infarct volume.

To improve interpretability and to create a modified NIHSS, the model was repeated using non-transformed variables and validated using bootstrapping techniques. The final regression equation was: DWI volume ($cm^3$) = 0.83 + (NIHSS)*(2.1) + (line cancellation % error)*(0.3) + (visual perception percent error)*(0.2).

### 3.3. Creation of NIHSS-Plus

When we converted the equation to total points, with the NIHSS component contributing up to 30 possible points, the line cancellation score contributed up to 4 additional points (percent error divided by 100 and multiplied by 4 would give the points for an individual) and the visual perception task contributed up to 3 additional points (percent error divided by 100 and multiplied by 3), for a maximum total combined score of 37. For example, if a patient had an NIHSS score of 12, 50% error rate on line cancellation (equivalent to 2 points), and 33% error on visual perception (equivalent to 1 point), his or her total NIHSS-plus score would be 15. Figure 1 shows an example of 2 subjects with different strokes but the same NIHSS score. Their NIHSS-plus scores represent the disparate size of their lesions more accurately. The NIHSS-plus and DWI volume had a Spearman's correlation (rho) of 0.54; this was higher than for the standard NIHSS and DWI volume (rho = 0.49).

## 4. Discussion

The NIHSS predicts DWI volume in this sample of nondominant stroke patients with a wide range of infarct sizes. However, the addition of a line cancellation and visual perception task significantly improve the model and improve prediction of DWI volume, which may more accurately represent stroke severity, at least for right hemisphere stroke. This may be because some patients with stroke have neglect or perceptual deficits, but no motor or sensory deficits. The existing NIHSS has only 2 points allotted for neglect, even though this

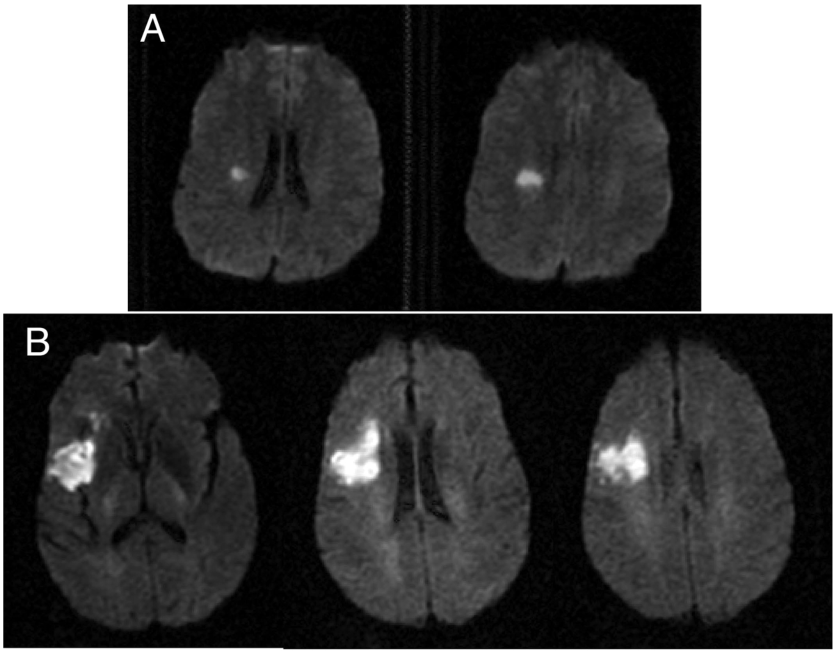

Fig. 1. DWI images from 2 patients (A and B). Both patients had an NIHSS of 3. Patient A would receive an NIHSS-plus of 3, and this patient's DWI volume is 1.03 cc. Patient B received an NIHSS-plus of 5.6, with a DWI volume measured at 24.2 cc.

deficit can be the most disabling consequence of right hemisphere stroke [14].

One of the primary limitations of our study relates to the extent of missing data. Although we excluded neglect tests with over 25% missing data, most of the tests included still had 15–20% missing data. Mean DWI volume among the patients with missing data on the two included tasks, compared with the mean DWI volume in subjects without missing data, showed no statistically significant difference in infarct size. In part because the entire neglect battery is time-consuming, some subjects either stop tolerating testing of any type or are interrupted for clinical tests or therapies.

This study may also be limited by its use of the retrospective assessment of the NIHSS (for some subjects). Although this technique has been validated [11], it is likely that it is not as accurate as a prospective assessment. However, in this study the NIHSS scores alone did show a similar correlation with infarct size as in previous studies [2,15].

Another potential limitation is the clinical utility of a measure that predicts infarct volume. A few authors have reported associations with DWI volume and functional scales (such as the Barthel Index) [16,17] but it will be important to determine that a clinical assessment of volume of ischemia truly corresponds to tangible patient outcomes. The NIHSS is least associated with functional outcomes in patients with cortical strokes [4]; it is likely that the NIHSS-plus would be more predictive than the NIHSS particularly for cortical strokes, as illustrated in Figure 1. In previous studies we have shown that line cancellation actually was more strongly associated with lesion volume and perfusion abnormality than was the NIHSS [7]. This result was not found in this study, probably because this study included many small strokes. Our previous study had relatively few small subcortical strokes, probably because of a referral bias. This study underscores that, contrary to conclusions drawn from studies of patients with persistent post-stroke deficits, most strokes are small.

A prospective study to validate the use of the NIHSS-plus in a separate sample of acute stroke patients (with both right and left hemisphere symptoms) will be critical to confirm that most patients are able to complete line cancellation and visual extinction tasks along with the NIHSS as a quick bedside assessment in the hyperacute setting. This will be particularly important in a sample of aphasic patients (left hemisphere strokes) because these tests may be difficult to interpret in this population. In addition, the scoring system could be simplified with further testing. In future prospective studies it will also be important to determine stroke location, because severity of symptoms and outcome likely depend on both size and location.

Numerous authors have proposed that cognitive measures be added to current means of assessment for stroke patients [18,19]. We propose that two simple cognitive tests (line cancellation and visual perception), which together can be completed in 2–3 minutes, should be combined with the NIHSS to create an "NIHSS-plus" that more accurately represents volume of ischemia in acute stroke. We have also obtained preliminary data in earlier research, indicating that one or two simple tests (oral naming, beyond that in the standard NIHSS, and repetition) [20] might be added to the NIHSS-plus, leading to a scale that represents not only motor dysfunction but more cognitive aspects of both left and right hemispheric dysfunction. A combined measure, using prospective administration of the NIHSS, line cancellation, visual perception, and oral naming (or repetition), could be validated in an independent sample of both left and right hemisphere stroke patients in the emergency setting, to determine if this NIHSS-plus represents the true extent of ischemic tissue. If proven reliable and valid, this NIHSS-plus could be used for assessment of baseline function and of outcome in stroke trials and in clinical practice.

## Disclosure/Conflict of Interest

The authors report no conflicts of interest.

## Acknowledgements

This research was supported by NIH grants K12 RR017627 (RG) and RO1-NS047691 (AH), and by a Johns Hopkins Clinician Scientist Award (RG).
